# Osthole Mitigates Progressive IgA Nephropathy by Inhibiting Reactive Oxygen Species Generation and NF-κB/NLRP3 Pathway

**DOI:** 10.1371/journal.pone.0077794

**Published:** 2013-10-28

**Authors:** Kuo-Feng Hua, Shun-Min Yang, Tzu-Yang Kao, Jia-Ming Chang, Hui-Ling Chen, Yung-Jen Tsai, Ann Chen, Sung-Sen Yang, Louis Kuoping Chao, Shuk-Man Ka

**Affiliations:** 1 Department of Biotechnology and Animal Science, National Ilan University, Ilan, Taiwan, Republic of China; 2 Department of Pathology, Tri-Service General Hospital, National Defense Medical Center, Taipei, Taiwan, Republic of China; 3 Department of Pharmacology, Institute for Drug Evaluation Platform, Development Center for Biotechnology, Taipei, Taiwan, Republic of China; 4 IND Core Team, Institute for Drug Evaluation Platform, Development Center for Biotechnology, Taipei, Taiwan, Republic of China; 5 Division of Nephrology, Department of Internal Medicine, Tri-Service General Hospital, National Defense Medical Center, Taipei, Taiwan, Republic of China; 6 Department of Cosmeceutics, China Medical University, Taichung, Taiwan, Republic of China; 7 Graduate Institute of Aerospace and Undersea Medicine, National Defense Medical Center, Taipei, Taiwan, Republic of China; Institut national de la santé et de la recherche médicale (INSERM), France

## Abstract

Renal reactive oxygen species (ROS) and mononuclear leukocyte infiltration are involved in the progressive stage (exacerbation) of IgA nephropathy (IgAN), which is characterized by glomerular proliferation and renal inflammation. The identification of the mechanism responsible for this critical stage of IgAN and the development of a therapeutic strategy remain a challenge. Osthole is a pure compound isolated from *Cnidiummonnieri* (L.) Cusson seeds, which are used as a traditional Chinese medicine, and is anti-inflammatory, anti-apoptotic, and anti-fibrotic both *in vitro* and *in vivo*. Recently, we showed that osthole acts as an anti-inflammatory agent by reducing nuclear factor-kappa B (NF-κB) activation in and ROS release by activated macrophages. In this study, we examined whether osthole could prevent the progression of IgAN using a progressive IgAN (Prg-IgAN) model in mice. Our results showed that osthole administration resulted in prevention of albuminuria, improved renal function, and blocking of renal progressive lesions, including glomerular proliferation, glomerular sclerosis, and periglomerular mononuclear leukocyte infiltration. These findings were associated with (1) reduced renal superoxide anion levels and increased Nrf2 nuclear translocation, (2) inhibited renal activation of NF-κB and the NLRP3 inflammasome, (3) decreased renal MCP-1 expression and mononuclear leukocyte infiltration, (4) inhibited ROS production and NLRP3 inflammasome activation in cultured, activated macrophages, and (5) inhibited ROS production and MCP-1 protein levels in cultured, activated mesangial cells. The results suggest that osthole exerts its reno-protective effects on the progression of IgAN by inhibiting ROS production and activation of NF-κB and the NLRP3 inflammasome in the kidney. Our data also confirm that ROS generation and activation of NF-κB and the NLRP3 inflammasome are crucial mechanistic events involved in the progression of the renal disorder.

## Introduction

Reactive oxygen species (ROS) play a major pathogenic role in a wide range of types of human [1] and experimental [2], [3] glomerulonephritis, including IgA nephropathy (IgAN) [4]–[7]. Importantly, in both patients [4], [5], [8] and animal models [7], [9], [10], ROS have been shown to be involved in the progressive stage of IgAN, the most common type of primary glomerulonephritis. In addition, mononuclear leukocyte infiltration in the kidney of IgAN patients is highly implicated in converting IgAN into progressive renal injury [11]–[13]. These inflammatory mediators and pathways are considered a key step in IgAN prior to the development of chronic renal failure [11], [12], [14], [15]. We previously established a progressive IgAN (Prg-IgAN) model in mice that is suitable for investigating the progression of IgAN [11] because of its clinical and pathological features of (1) a rapid decline in renal function, (2) greatly enhanced glomerular proliferation, and (3) periglomerular mononuclear leukocyte infiltration associated with accelerated activation of the transcription factor, nuclear factor-kappa B (NF-κB). All these pathological features are often seen in Prg-IgAN patients [14], [16]. However, the precise mechanism responsible for progression of IgAN remains to be determined and its identification might help in the development of a therapeutic strategy.

Osthole is a pure compound isolated from *Cnidiummonnieri* (L.) Cusson seeds, which are used as a traditional Chinese medicine, and has been shown to have an anti-inflammatory effect in murine macrophages [17], attenuates experimental autoimmune encephalomyelitis and acute lung injury in mice [18], [19], reduces acute ischemic stroke and ischemia-reperfusion induced renal injury in rat [20], [21], an anti-apoptotic effect in a mouse model of inflammation [22], and anti-fibrotic effects *in vitro* [23] and *in vivo* [24]. However, the effects of osthole on IgAN and relevant clinical trials have not been reported yet. We previously showed that osthole reduces NF-κB activation and ROS release inactivated macrophages [17]. All these prompted us to test the hypothesis that osthole might prevent the progressive renal injury associated with glomerular proliferation and renal inflammation in IgAN.

In the present study, we demonstrated that osthole had a beneficial effect in a mouse Prg-IgAN model by reducing superoxide anion production and inhibiting NF-κB and nacht domain-, leucine-rich repeat-, and pyrin domain-containing protein 3 (NLRP3) inflammasome activation *in vivo* and *in vitro*.

## Materials and Methods

### Ethic Statement

All animal experiments were performed with the ethical approval of the Institutional Animal Care and Use Committee of The National Defense Medical Center, Taiwan and according to the ethical rules in the NIH *Guide for the Care and Use of Laboratory Animals*. The animals were maintained in the Animal Center of the National Defense Medical Center (Taipei, Taiwan).

### Extraction and Purification of Osthole

Osthole, 7-methoxy-8-(3-methyl-2-butenyl) coumarin, was purified from *Cnidiummonnieri* (L.) Cusson seeds as described in our previous work [17]. Briefly, the extracts obtained from *C. monnieri*seeds that were treated with ethanol at room temperature were concentrated to produce alcoholic extracts (AE), and was dissolved in ethanol, followed by semipreparative high-performance liquid chromatography for further purification. Among the five pure compounds obtained, osthole was identified as colorless prisms from ether; mp 79–81°C; EI-MS *m/z*(%) 244 (M^+^, 100), 229 (38), 213 (23), 201 (28), 189 (38), 131 (25); UV MeOHλ_max_ 249, 258, 322; IR KBrλ_max_ (cm^−1^) 1720, 1610; ^1^HNMRδ 7.61 (1 H, d, *J* = 9.4 Hz), 7.29 (1 H, d, *J* = 8.7 Hz), 6.83 (1 H, d, *J* = 8.7 Hz), 6.23 (1 H, d, *J* = 9.4 Hz), 5.22 (1 H, t, *J* = 7.4 Hz), 3.92 (3 H, s), 3.52 (2 H, d, *J* = 7.4 Hz), 1.84 (3 H, s), 1.67 (3 H, s).

### Prg-IgANmodel and Experimental Protocol

Experiments were performed on 8-week-old female B cell deficiency (BCD) (B6.129S2-Igh-6tm1Cgn/J) mice obtained from Professor John T. Kung (Institute of Molecular Biology, Academia Sinica, Taipei, Taiwan). IgAN was induced by 28 daily injections of purified IgA anti-phosphorylcholine antibodies and pneumococcal C-polysaccharide (PnC), and this model features absence of IgG and IgM, and lower C3 deposits than its wild type mice, as described previously [11], [12]. Based on the previous studies [21], [25], [26] the effective dose of osthole is around 20–40 mg/kg, therefore we treated the mice with osthole at concentration of 30 mg/kg. Starting three days before the beginning of IgAN induction on day 0, groups of mice (n = 7 each) were injected intraperitoneally with a daily dose of osthole (30 mg/kg body weight) or vehicle (mixture of DMF, Tween-80, and saline) until day 28 (32 days) after the start of IgAN induction, then the mice were sacrificed. Age- and sex-matched BCD mice which received daily saline injections were used as normal controls. Body weight was measured weekly and urine samples were collected in metabolic cages on days 3, 7, 14, 21, and 28. Renal cortical tissues and blood samples were collected and stored appropriately for further analysis. The concentration of urine albumin was determined by ELISA (Exocell, Philadelphia, PA) and urine albumin levels expressed relative to urine creatinine (Cr) levels measured using a kit from Wako Pure Chemical Industries (Osaka, Japan) as described previously [12]. Serum levels of blood urea nitrogen (BUN) and Cr were measured using BUN kits or Cr kits (both from Fuji Dry-Chem Slide, Fuji Film Medical, Tokyo, Japan), as described previously [27].

### Pathologic Evaluation

For renal pathological evaluation, the renal tissues were fixed in 10% buffered formalin, embedded in paraffin, and 4 µmsections prepared and stained with hematoxylin and eosin (H&E) as described previously [27]. Renal pathology was examined and renal lesions scored as described previously [27]. The percentage of glomeruli showing glomerular proliferation, glomerular sclerosis, or periglomerular inflammation in one hundred glomeruli in at least two renal tissue sections per slide was calculated under light microscopy at a magnification of x400. The severity of renal lesions was scored as described previously [28]. For immunofluorescence (IF) FITC-conjugated goat anti-mouse IgA and C3 (Cappel; OrganonTeknika, Durham, NC) were employed and a semiquantitative evaluation was performed as described previously [28]. For immunohistochemical staining for phosphorylated NF-κB p65 (pNF-κB p65), collagen IV (Col-IV), or TGF-β, formalin-fixed and paraffin-embedded renal sections were incubated with primary antibodies against mouse pNF-κB p65 (Cell Signaling Technology, MA), Col-IV (Southern Biotech, AL), or TGF-β (Santa Cruz, CA), then with biotinylated secondary antibodies and avidin-biotin-peroxidase complex (both from Dako, Glostrup, Denmark Dako) as described previously [27]. Quantitative image analysis software (Pax-it; Paxcam, Villa Park, IL) was used to score staining for pNF-κB p65 by counting the number of stained cells and to score staining for Col-IV and TGF-β by quantifying the signal intensity in 20 glomeruli as the positive area expressed as a percentage of the area of the entire glomerulus as described previously and twenty randomly selected fields of tubulointerstitial compartment in the cortical area were examined at the magnification of ×400 by light microscopy and expressed as cells per field [27]. For immunohistochemical staining for CD3^+^ (pan-T cells) or F4/80^+^ (macrophages), methyl Carnoy’s solution-fixed, paraffin-embedded renal sections were stained with biotin-conjugated antibodies against mouse CD3^+^ or F4/80^+^ (Serotec, Oxford, UK), respectively. For detection of apoptosis, formalin-fixed tissue sections were stained using a TUNEL staining kit (ApopTagPlus Peroxidase In Situ Apoptosis Detection kit; Chemicon, Temecula, CA) according to the manufacturer’s instructions. Numbers of CD3-, F4/80-, or TUNEL-positive cells in 20 glomeruli were counted using Pax-it software.

### Superoxide Anion Measurement

Superoxide anion levels in kidney tissues were measured as described previously [29] and the results expressed as reactive luminescence units (RLU) per 15 min per mg dry weight of tissue (RLU/15 min/mg dry weight).

### Western Blot Analysis of Nuclear Factor E2-related Factor 2 (Nrf2), NLRP3, and Caspases

Cytoplasmic and nuclear proteins were extracted from renal tissues using a nuclear extract kit (Active Motif, Tokyo, Japan) according to the manufacturer’s instructions and target proteins detected by SDS-PAGE electrophoresis and immunoblotting using rabbit antibodies against mouse Nrf2 (Santa Cruz), NLRP3 (Santa Cruz), caspase-1 (US Biological, MA), caspase-3 or caspase-9 (Cell Signaling) and horseradish peroxidase (HRP) -conjugated goat anti-rabbit IgG antibodies (Dako) as described previously [27]. Antibodies to lamin A or β-actin (Santa Cruz) were used as internal controls for the nuclear and cytosolic proteins, respectively.

### Measurement of Cellular Heme Oxidase-1 (HO-1) Level and Glutathione Peroxidase (GPx) Activity

HO-1 level (ELISA kit, R&D Systems, MN) or GPx activity (assay kit, Cayman, MI) in renal tissue was measured according to the manufacturer’s instructions.

### *In vitro* Experiments

#### Macrophages

Murine macrophage cell line J774A.1 was purchased obtained from the American Type Culture Collection (Rockville, MD). The cells were cultured in RPMI 1640 medium supplemented with 10% heat-inactivated fetal calf serum and 2 mM L-glutamine (all from Life Technologies, Carlsbad, CA) at 37°C in a 5% CO_2_ incubator. In tests involving sequential additions, e.g. pretreatment with inhibitors, unless otherwise stated, all reagents present in the previous step were still present during the next step.

For ELISA, cells (2 × 10^6^ in 2 ml of medium) were seeded in 6-well plates and treated as indicated conditions. Secretion of IL-1β was measured according to the manufacturer’s protocol (eBioscience, CA). In brief, 50 µl of biotinylated antibody and 50 µl of supernatant were added to a strip well plate precoated with anti-mouse IL-1β antibodies and incubated at room temperature for 2 h. After three washes with washing buffer, 100 µl of diluted HRP-conjugated streptavidin concentrate was added to each well and the plate incubated at room temperature for 30 min. The washing process was repeated, then 100 µl of a premixed tetramethylbenzidine substrate solution was added to each well and the reaction developed at room temperature in the dark for 30 min. Following addition of 100 µl of stop solution to each well, the absorbance was read in a microplate reader at 450 nm.

For Western blotting, after treated the cells with the indicated condition, then the cells were collected and lysed at 4°C in lysis buffer (25 mM Tris-HCl, pH 7.5, 100 mM NaCl, 2.5 mM EDTA, 2.5 mM EGTA, 20 mM NaF, 1 mM Na3VO4, 20 mM sodium β-glycerophosphate, 10 mM sodium pyrophosphate, 0.5% Triton X-100) containing protease inhibitor cocktail (Sigma, St. Louis, MO), then the whole cell lysate was separated by SDS-PAGE and electrotransferred to a PVDF membrane. The membranes were incubated for 1 h at room temperature in blocking solution (5% nonfat milk in phosphate buffered saline with 0.1% Tween 20), then were incubated for 2 h at room temperature with rabbit antibodies against mouse β-actin, NLRP3, caspase-1 p10, phosphorylated PKC-α (all from Santa Cruz) or phosphorylated p38 antibody (Sigma) in blocking solution. After three washes in PBS with 0.1% Tween 20, the membrane was incubated for 1 h at room temperature with HRP-conjugated goat anti rabbit secondary antibody in blocking buffer and developed using an enhanced chemiluminescence Western blot detection system.

Intracellular production of ROS stimulated by ATP was measured by detecting the fluorescence intensity of 2′,7′-dichlorofluorescein diacetate oxidized product (Molecular Probes, Eugene, OR). Briefly, J774A.1 macrophages (1×10^6^ in 1 ml of medium) grown in a phenol red-free RPMI medium for 24 h, then primed for 6 h with 1 µg/ml of LPS, then for 30 min with 50 µM of osthole or 10 mM of NAC at 37°C in the dark in the presence of 2 µM of 2′,7′-dichlorofluorescein diacetate, then for 0–40 min with or without addition of 5 mM ATP. The relative fluorescence intensity of oxidized product of 2′,7′-dichlorofluorescein diacetate, 2′,7′-dichlorofluorescein, was detected at an excitation wavelength of 485 nm and an emission wavelength of 530 nm with a CytoFluor 2300 fluorometer (Millipore, Bedford, MA).

#### Mesangial cells

For ROS production assay, murine mesangial cells CRL 1927 (1×10^6^ in 1 ml of medium) grown in a phenol red-free RPMI medium for 24 h, then incubated for 30 min with 50 µM of osthole or 10 mM of NAC at 37°C in the dark in the presence of 2 µM of 2′,7′-dichlorofluorescein diacetate, then for 0–80 min with or without addition of 2 µg/ml LPS, and then ROS production was detected as described in macrophages. For MCP-1 production assay, CRL 1927 cells (3×10^4^ in 1 ml of medium) were incubated for 2 h with 0–50 µM of osthole, then for 24 h with or without addition of 2 µg/ml LPS, and then MCP-1 concentration in conditional medium were measured by ELISA. Next, murine mesangial cells (4×10^5^ in 5 ml of medium) were incubated for 2 h with osthole, then for 24 h with or without addition of 2 µg/ml LPS, and nuclear protein from the cells were extracted described as above. The protein levels of nuclear NF-κB p65 were determined by an ELISA-based TransAM NF-κB kit (Active Motif, Tokyo, Japan) according to the manufacturer’s instructions. The protein levels of nuclear Nrf2 were evaluated by Western blotting with a rabbit antibody against mouse Nrf2 (Santa Cruz).

### Data Analysis

For *in vivo* experiments, the results are presented as the mean ± SEM. Comparisons between two groups were performed using Student’s *t* test. The significance of differences in urinary albumin/creatinine levels were examined using one-way ANOVA, with post hoc correction by Tukey’s method. For *in vitro* experiments, all values are given as mean ± SD. Data analysis involved one-way ANOVA with a subsequent Scheffe’ test. A *p* value *<*0.05 was considered statistically significant.

## Results

### Osthole Ameliorated the Progressive Stage of IgAN in Mice

#### Albuminuria, renal function, and renal injury

**Figure 1:**
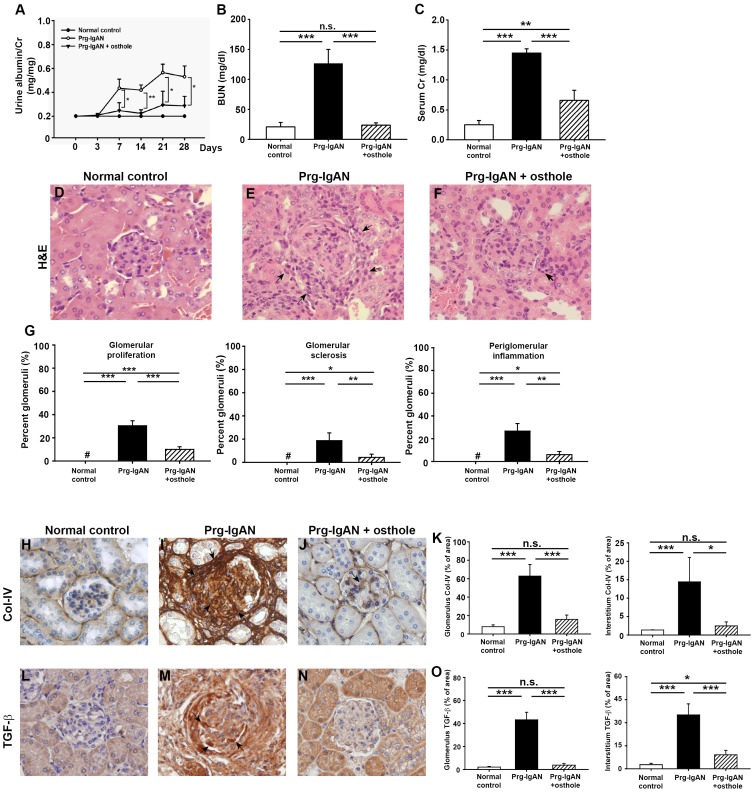
Urine albumin, renal function, and renal pathology. Time-course study of urine albumin levels on days 3, 7, 14, 21, and 28 (A). Serum blood urea nitrogen (BUN) levels (B), and serum creatinine (Cr) levels (C) on day 28. Kidney histopathology on day 28 shown by H&E staining (D–G). The arrowheads in E and F indicate peri-glomerular mononuclear leukocyte infiltration. Detection of collagen IV (Col-IV) (H–K) and TGF-β (L–O) by IHC staining. The arrowheads in the stained panels indicate positive staining. Original magnification, 400×. In the histograms, n = 7, *p<0.05, **p<0.01, ***p<0.005. #, not detectable, and n.s. not significant.

Prg-IgAN mice treated with vehicle showed a significant increase in urine albumin levels beginning on day 7 after disease induction and levels continued to increase until day 28 when the animals were sacrificed (Fig. 1A). Osthole treatment of Prg-IgAN mice (Prg-IgAN+osthole) resulted in greatly decreased urine albumin levels compared to Prg-IgAN mice (p<0.05). Prg-IgAN mice showed impaired renal function, as demonstrated by significantly higher levels of serum BUN (Fig. 1B) and Cr (Fig. 1C) on day 28 (both p<0.005), while Prg-IgAN+osthole mice showed improved renal function, with decreased serum BUN and Cr levels. Prg-IgAN+osthole mice also had significant higher serum Cr levels than normal control mice (p<0.01).

As shown in Fig. 1D–G, Prg-IgAN mice showed marked glomerular proliferation, mainly mesangial cells and focal, but intense, glomerular sclerosis, as well as interstitial (mainly periglomerular) mononuclear leukocyte infiltration on day 28 and these pathological lesions were markedly reduced in the Prg-IgAN+osthole mice. By IF staining, there was no significant difference in the intensity of IgA and C3 deposition in the glomerulus between the Prg-IgAN and Prg-IgAN+osthole mice (data not shown), indicating that treatment with osthole had no effect on immune complex deposition. In addition, expression of Col-IV (Fig. 1H–K) and TGF-β Fig. 1L–O) was significantly increased in Prg-IgAN mice compared to normal control mice by immunohistochemistry (*p*<0.01), and these effects were substantially decreased in the Prg-IgAN+osthole mice (*p*<0.05).

The changes in mice body weight of each group were no significant different (normal control group: 19.1±0.5 g; Prg-IgAN group: 18.5±0.6 g; Prg-IgAN+osthole group: 18.9±0.5 g). In addition, the condition of the mice did not decline significantly during the experiment and no significant hair loss or appetite change was observed.

#### Renal superoxide anion levels and Nrf2 nuclear translocation

**Figure 2:**
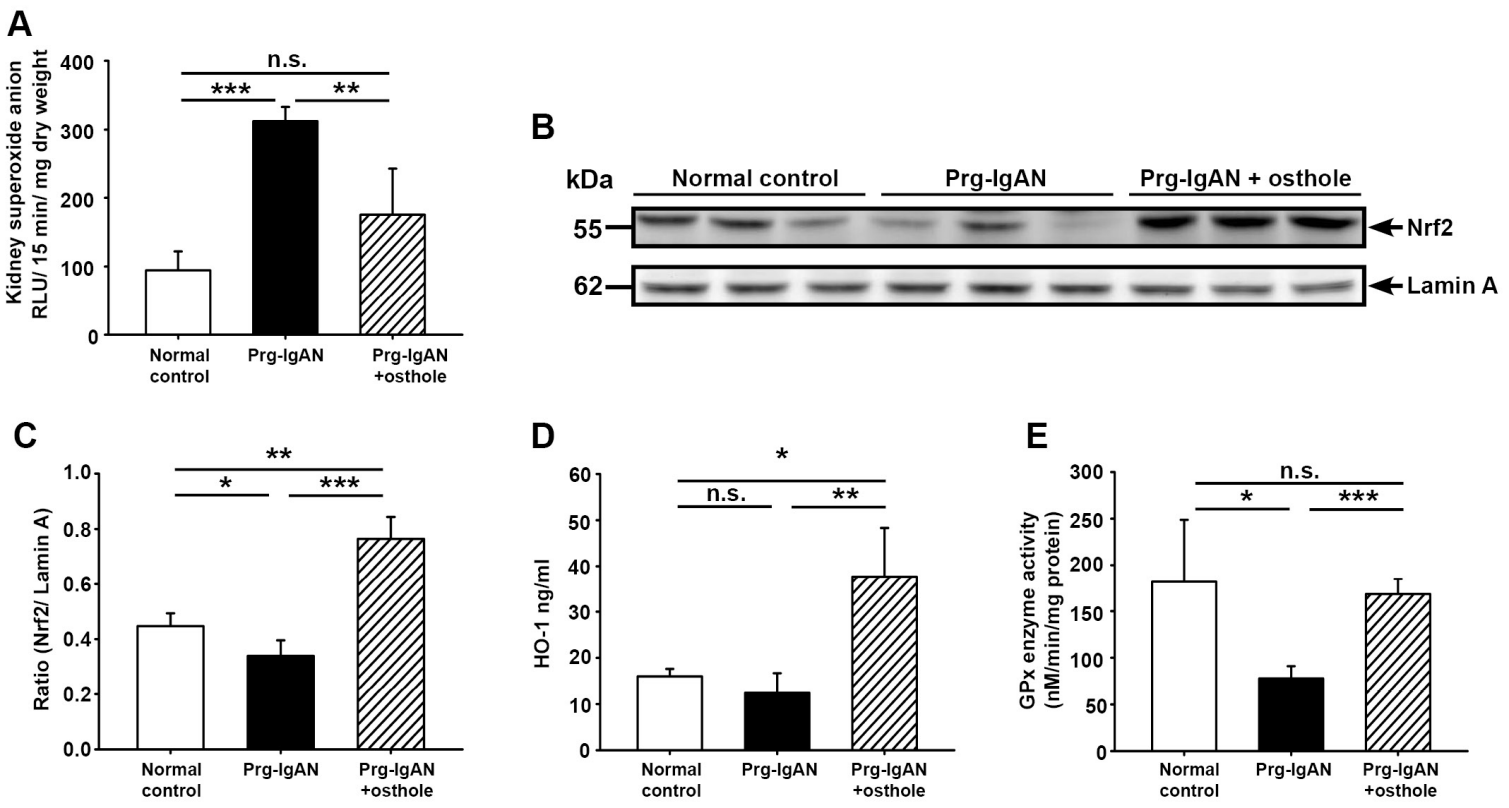
Renal superoxide anion levels, Nrf2 nuclear translocation, and cytoplasmic HO-1 levels and GPx activity in renal tissues. Renal superoxide anion levels (A). Representative Western blots (B) for nuclear Nrf2 with lamin A as the internal control and the Nrf2/laminA ratio (C). Renal cytosolic HO-1 levels (D). Renal cytosolic GPx activity (E). In the histograms, n = 7, *p<0.05, **p<0.01, ***p<0.005, and n.s. not significant.

At 28 days after the start of disease induction, Prg-IgAN mice had significantly higher renal levels of superoxide anion than normal control mice (Fig. 2A; *p*<0.005) and this effect was significantly reduced in the Prg-IgAN+osthole mice (*p*<0.01). Nrf2 is known to exert its beneficial effects on renal conditions by counteracting oxidative stress [30]. We therefore measured renal protein nuclear Nrf2 (active Nrf2) levels and cytosolic HO-1 levels and GPx activity (the last two being involved in the Nrf2 pathway). Compared to both Prg-IgAN mice and normal controls, Prg-IgAN+osthole mice had significantly higher nuclear levels of Nrf2 protein (*p*<0.01) (Fig. 2B, C). In parallel, cytosolic HO-1 expression was much higher in the Prg-IgAN+osthole mice than either the Prg-IgAN mice or the normal control mice (*p*<0.05) (Fig. 2D). Prg-IgAN mice had significantly lower GPx activity than normal control mice (*p*<0.05) and this effect was markedly inhibited by osthole treatment (*p*<0.005) (Fig. 2E).

#### Renal activation of NF-κB and the NLRP3 inflammasome

**Figure 3:**
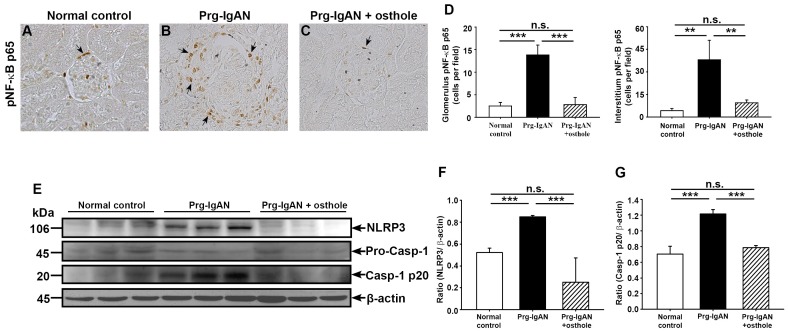
Renal NF-κB p65 nuclear translocation and NLRP3 inflammasome activation. Detection of phosphorylated NF-κB p65 (pNF-κB p65) by immunohistochemisty staining (A–D). The arrowheads indicate positively stained cells. Original magnification, 400×. Representative Western blots for renal NLRP3, procaspase-1, and active caspase-1 with β-actin as the internal control (E) and the NLRP3/β-actin ratio (F) and active caspase-1/β-actin ratio (G). In the histograms, n = 7, **p<0.01, ***p<0.005, and n.s. not significant.

Activation of the NF-κB pathway has been implicated in the acceleration and progression of IgAN [16], [31]. As shown in Fig. 3A–D, at day 28 after the start of disease induction, the Prg-IgAN mice showed significantly increased renal nuclear translocation of pNF-κB p65 compared to normal control mice (*p*<0.005) and this effect was markedly inhibited in the Prg-IgAN+osthole mice (*p*<0.005). The NLRP3 inflammasome activates caspase-1, leading to IL-1β and IL-18 production [32]. As shown on Western blots, at day 28 after the start of disease induction, increased renal NLRP3 protein levels were observed in Prg-IgAN mice compared to normal control mice (*p*<0.005) and osthole treatment resulted in a significant reduction in levels (*p*<0.005) (Fig. 3E, F). Prg-IgAN mice also showed significantly higher levels of mature caspase-1 than normal control mice (*p*<0.005) and osthole administration resulted in a significant decrease in levels (*p*<0.005) (Fig. 3E, G).

#### Renal MCP-1 expression and mononuclear leukocyte infiltration

**Figure 4:**
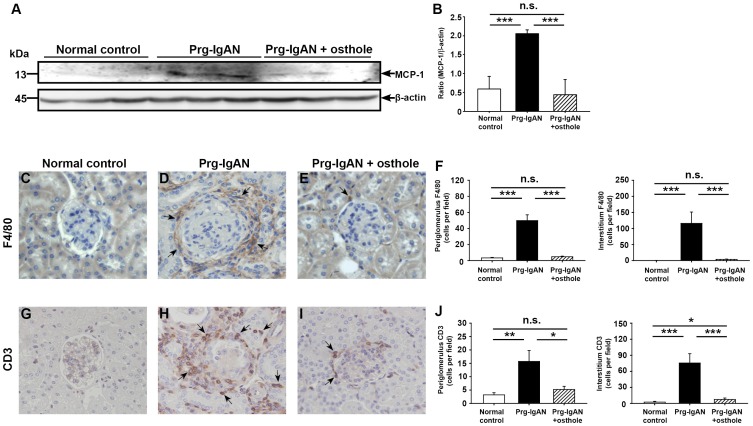
Renal MCP-1 expression and infiltration of monocytes/macrophages and T cells. Representative Western blots of cytosolic MCP-1 in renal tissues, with β-actin as the internal control (A). MCP-1/β-actin ratio (B). Detection of F4/80^+^ monocytes/macrophages (C–F) and CD3^+^ T cells (G–J) by immunohistochemistry. Original magnification, 400×. The arrowheads indicate positively stained cells. In the histograms, n = 7, *p<0.05, **p<0.01, ***p<0.005, and n.s. not significant.

As seen in Fig. 4A, B, Western blots showed that, on day 28 after the start of disease induction, Prg-IgAN mice had significantly higher renal MCP-1 levels than normal control mice (p<0.005) and this effect was markedly inhibited in Prg-IgAN+osthole mice (p<0.005). As shown in Fig. 4C–F, significant renal periglomerular infiltration of macrophages (F4/80^+^) was seen in Prg-IgAN mice compared to normal control mice (p<0.005) and this effect was also markedly inhibited in Prg-IgAN+osthole mice (p<0.005). In addition, as shown in Fig. 4G–J, Prg-IgAN mice showed significantly increased periglomerular infiltration of T cells (CD3^+^) in the kidney compared to normal controls (p<0.005) and this effect was markedly reduced in Prg-IgAN+osthole mice (p<0.01). There was no detectable difference in periglomerular infiltration of both cell types between Prg-IgAN+osthole and normal control mice.

#### Renal apoptosis

**Figure 5:**
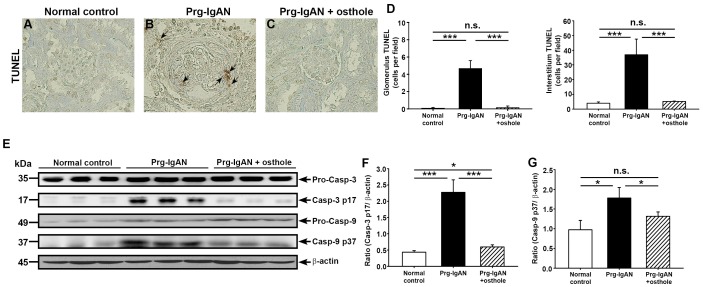
Renal apoptosis in the glomerulus and tubule. TUNEL staining of renal tissues at day 28 (A–D). The arrowheads indicate positively stained cells, Original magnification, 400×. Representative Western blots for renal pro-caspase-3, pro-caspase-9, and their active forms, with β-actin as the internal control (E) and the active caspase-3/β-actin ratio (F) and active caspase-9/β-actin ratio (G). In the histograms, n = 7, *p<0.05, ***p<0.005, and n.s. not significant.

As shown by TUNEL staining (Fig. 5A–D), the Prg-IgAN mice showed greatly increased renal apoptosis at 28 days after the start of disease induction compared to normal control mice (*p<*0.01) and this effect was significantly inhibited in Prg-IgAN+osthole mice (*p<*0.01). As shown in Fig. 5E, F, when renal levels of activated caspase-3 and caspase-9 were examined, levels of the mature form (p17 fragment) of caspase-3 were greatly increased in Prg-IgAN mice compared to normal control mice (*p<*0.005) and this effect was markedly inhibited in Prg-IgAN+osthole mice (*p<*0.005). In addition, as shown in Fig. 5E and G, levels of the mature form (p37 fragment) of renal caspase-9 were increased in Prg-IgAN mice (*p*<0.05) and this effect was again markedly reduced in Prg-IgAN+osthole mice (*p*<0.05).

### Osthole Inhibited Inflammatory Responses *in vitro*

#### Macrophages

**Figure 6:**
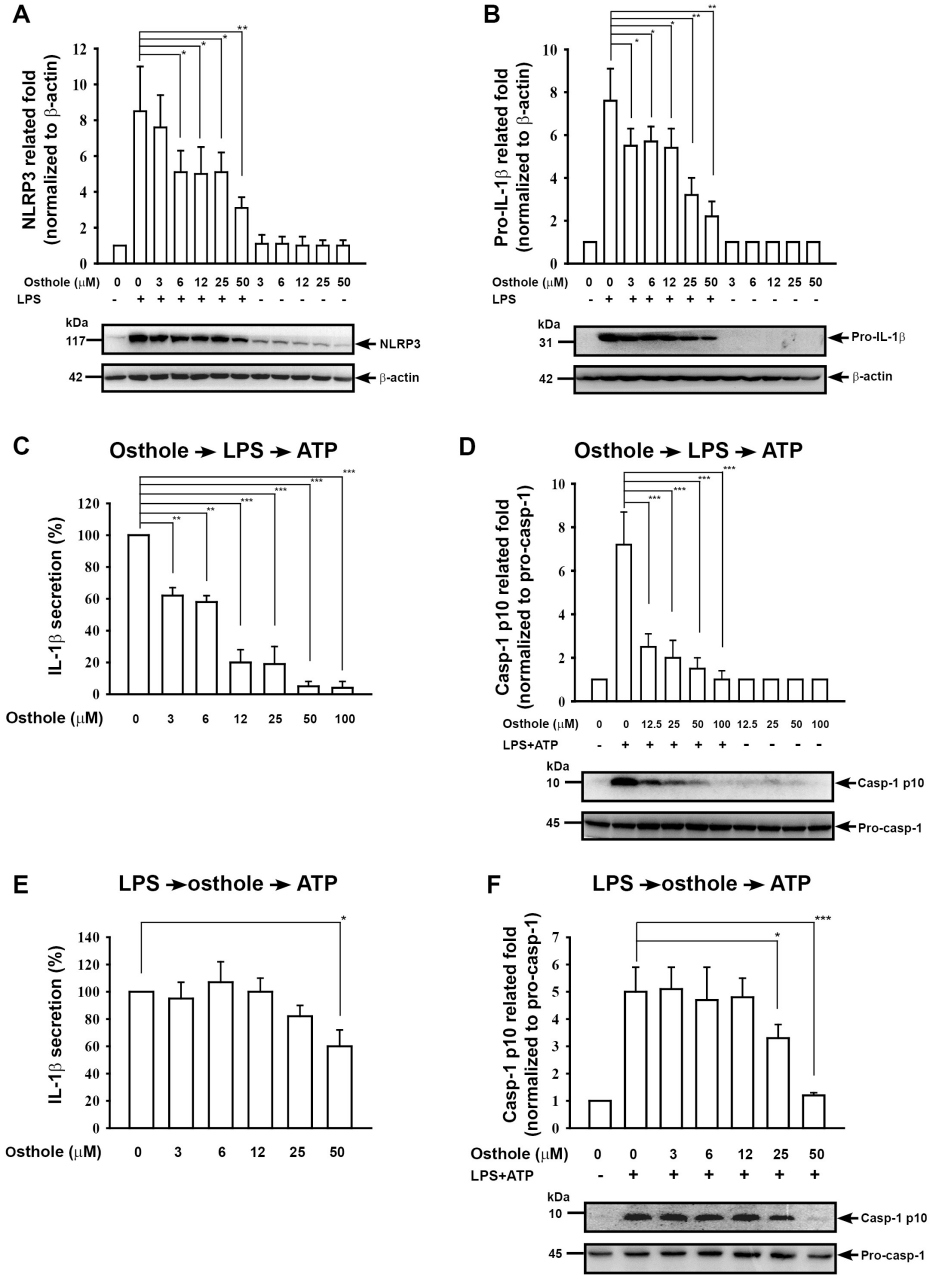
Effect of osthole on NLRP3 inflammasome activation. (A) and (B) J774A.1 macrophages (2×10^6^ in 2 ml of medium) were incubated for 30 min with or without the indicated concentration of osthole and for 6 h with or without addition of 1 µg/ml of LPS, then expression of NLRP3 (A) and pro-IL-1β (B) was analyzed by Western blotting. (C) and (D) J774A.1 macrophages (2×10^6^ in 2 ml of medium) were incubated for 30 min with or without the indicated concentration of osthole, then for 6 h with or without addition of 1 µg/ml of LPS. After washing, the cells were incubated with or without 5 mM ATP for 30 min, then IL-1β in the culture medium was measured by ELISA (C) and active caspase-1 (p10) and caspase-1 (p45) in the cells measured by Western blotting (D). (E) and (F) J774A.1 macrophages (2×10^6^ in 2 ml of medium) were incubated for 6 h with or without 1 µg/ml of LPS, then for 30 min with or without addition of the indicated concentration of osthole, followed by 30 min incubation with or without addition of 5 mM ATP, then IL-1β in the culture medium were measured by ELISA (E) and active caspase-1 (p10) and caspase-1 (p45) in the cells were measured by Western blotting (F). (C) and (E) the data are expressed as the mean ± SD for three separate experiments, while, in (A), (B), (D), and (F), the results are representative of those obtained in three different experiments and the histogram shows the results for all 3 experiments expressed as the mean ± SD. *p<0.05, **p<0.01, ***p<0.005.

NLRP3 inflammasome activation and IL-1β secretion. Full activation of the NLRP3 inflammasome requires both a priming signal from pathogen-associated molecular patterns, e.g., LPS, the major cell wall component of gram-negative bacteria, and an activation signal from a second stimulus, e.g. damage-associated molecular patterns, the former controlling the expression of NLRP3 (an essential component of the NLRP3 inflammasome) and pro-IL-1β (the precursor of IL-1β) and the latter controlling caspase-1 activation [33]. First, we investigated whether osthole was able to inhibit protein expressions of NLRP3 protein and pro-IL-1β in LPS-activated macrophages. The results showed that the LPS-induced increase in NLRP3 (Fig. 6A) and pro-IL-1β (Fig. 6B) protein levels was inhibited by osthole (*p*<0.05). We then examined whether osthole was able to inhibit NLRP3 inflammasome activation by affecting LPS-mediated priming signal. When the cells were incubated with osthole for 30 min before LPS priming, the osthole and LPS washed out, and ATP added for another 30 min before measuring IL-1β secretion and caspase-1 activation, IL-1β secretion (Fig. 6C) and caspase-1 activation (Fig. 6D) were significantly inhibited by osthole in a dose-dependent fashion (*p*<0.01). Furthermore, we investigated whether osthole was able to inhibit NLRP3 inflammasome activation by affecting ATP-mediated activation signal. ATP induced IL-1β secretion (Fig. 6E) and caspase-1 activation (Fig. 6F) in LPS-primed cells (*p*<0.05). Both effects were inhibited by pre-incubation of the cells with the higher concentrations (IL-1β: 50 µM; Caspase-1: 25 and 50 µM, respectively) of osthole before, and during ATP, stimulation. These data suggest that osthole inhibits NLRP3 inflammasome activation by acting on both the ATP- and LPS-mediated signaling stages.

**Figure 7:**
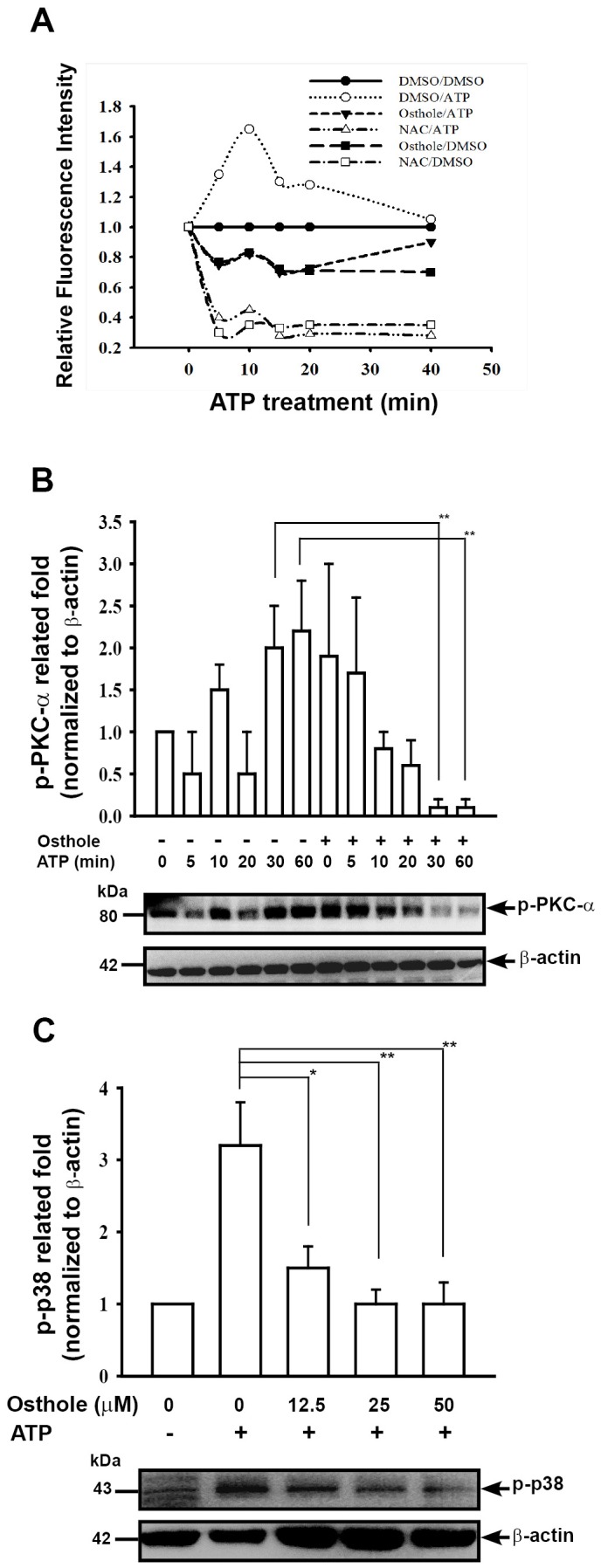
Effect of osthole on ATP-induced ROS production and PKC-α and p38 phosphorylation. (A) J774A.1 macrophages (1×10^6^ in 1 ml of medium) were incubated for 6 h with 1 µg/ml of LPS, then for 30 min with or without addition of 50 µM osthole or 10 mM NAC, then for 0–40 min with or without addition of 5 mM ATP. ROS production was measured as the relative fluorescence intensity, as described in the Materials and Methods. The data are expressed as the mean ± SD for three separate experiments. (B) J774A.1 macrophages (2×10^6^ in 2 ml of medium) were incubated for 6 h with 1 µg/ml of LPS, then for 30 min with or without addition of 50 µM osthole, then for 0–60 min with or without addition of 5 mM ATP, then phosphorylation of PKC-α was measured by Western blotting. (C) J774A.1 macrophages (2×10^6^ in 2 ml of medium) were incubated for 6 h with 1 µg/ml of LPS, then for 30 min with or without the indicated concentration of osthole, then for 30 min with or without addition of 5 mM ATP, then phosphorylation of p38 was measured by Western blotting.*p<0.05, **p<0.01.

ROS production and phosphorylation levels of PKC-α and p38. ROS play important roles on NLRP3 inflammasome activation [34]. In our previous study, we demonstrated that LPS-induced increase in ROS levels of macrophages was inhibited by osthole [17]. We then examined whether osthole was able to inhibit ATP-induced ROS production. The ATP-induced increased levels in ROS in LPS-primed cells was reduced by incubation with osthole and the potent antioxidant NAC 30 min before, and during, ATP stimulation (Fig. 7A). To examine whether other signaling pathways induced by ATP were affected by osthole, the LPS-primed cells were pre-incubated with osthole for 30 min, then were subjected to ATP stimulation. The results indicated that osthole inhibited the ATP-induced increase in the phosphorylation levels of PKC-α (Fig. 7B) and p38 (Fig. 7C) in LPS-primed macrophages (*p*<0.05).

#### Mesangial cells

**Figure 8:**
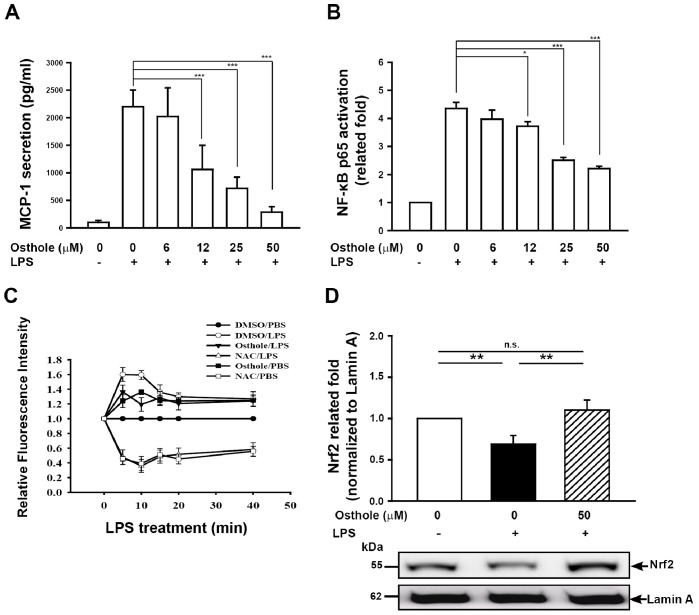
Effects of osthole on LPS-induced MCP-1 secretion, ROS generation, and activation of NF-κB and Nrf2 in mesangial cells. (A) Mesangial cells (3×10^4^ in 1 ml of medium) were incubated for 2 h with or without osthole as indicated, then for 24 h with or without addition of 2 µg/ml LPS. MCP-1 concentration in conditional medium was measured by ELISA. (B) pNF-κB p65 activation was measured by an ELISA-based activity assay. (C) Mesangial cells (1×10^6^ in 1 ml of medium) were incubated for 30 min with or without 50 µM osthole or 10 mM NAC, then for 0–40 min with or without addition of 2 µg/ml LPS. ROS production was measured as the relative fluorescence intensity, as described in the Materials and Methods. (D) Representative Western blots for nuclear Nrf2 with lamin A as internal control. *p<0.05, **p<0.01, ***p<0.005.

MCP-1 levels and pNF-κB activity. Mesangial cells are considered to play the leading role in the progression of IgA nephropathy. It has been demonstrated that mesangial cells can produce MCP-1, which attracts macrophages to the kidney in IgA nephropathy [35], [36]. We then evaluated the effect of osthole on the production of MCP-1 in LPS-treated mesangial cells. The results showed that greatly increased levels of MCP-1 were seen in LPS-treated mesangial cells compared to normal control, and this effect was significantly suppressed by osthole administration in a dose-dependent manner (*p*<0.005) (Fig. 8A). In addition, LPS-treated mesangial cells showed significantly higher pNF-κB p65 activity than normal control and this effect was greatly inhibited in the cell culture with osthole in a dose-dependent manner (*p*<0.05) (Fig. 8B).

ROS production and nuclear Nrf2 protein levels. ROS production in the mesangial area of the glomerulus has been shown to play a pathogenic role in the pathogenesis of IgAN [5]. We examined whether osthole was able to inhibit ROS production in LPS-treated mesangial cells. The LPS-induced ROS production in mesanigal cells was significantly reduced by incubation with osthole (*p*<0.005) (Fig. 8C). Besides, nuclear Nrf2 protein levels were greatly reduced in LPS-treated mesangial cells compared to those of normal control, this effect was significantly inhibited by osthole administration (*p*<0.01) (Fig. 8D).

## Discussion

In the present study, we showed that osthole can ameliorate the renal lesions of glomerular proliferation, sclerosis, and periglomerular infiltration of macrophages and T cells that are indicative of the progressive stage of IgAN in the mouse model. Our data suggest that the beneficial effects of osthole on Prg-IgAN mice were mainly through inhibiting ROS generation and NF-κB activationas well as reduced inflammatory cytokine expression and NLRP3 inflammasome activation in the kidney.

One mechanism by which osthole exerted its beneficial effects in preventing the development of progressive renal lesions in Prg-IgAN mice could be inhibition of ROS production, as demonstrated by the fact that osthole administration significantly inhibited the increase in superoxide anion levels in renal tissues seen in Prg-IgAN mice (Fig. 2A). This is consistent with our previous finding that osthole markedly inhibits ROS production by LPS-activated macrophages [17]. Activation of the Nrf2-mediated antioxidant pathway is a cellular defense response against ROS [37], [38]. In the present study, we confirmed this link, as demonstrated by increased nuclear translocation of Nrf2 protein, HO-1 levels, and GPx activity (the last two being involved in the Nrf2 pathway) in renal tissues in Prg-IgAN+osthole mice compared to Prg-IgAN mice. These results support the idea that osthole acts by decreasing ROS production and increasing activation of the NrF2 anti-oxidant pathway, resulting in attenuated renal pathology in osthole-treated mice.

In addition, in both patients [39]–[41] and animal models [42], [43], IL-1β plays a pathogenic role in the evolution of IgAN, while patients with IgAN have been shown to have increased IL-18 levels in urine [44] and serum [45] and this is considered as a prognostic factor in these patients [45]. Mature IL-1β and IL-18 are generated from their respective precursors, pro-IL-1β and pro-IL-18, by active caspase-1 produced by the NLRP3 inflammasome [32], [46]. Vilaysane A *et al.* [47] demonstrated that renal inflammation in a mouse renal fibrosis model is associated with activation of the NLRP3 inflammasome and overproduction of IL-1β and IL-18, and that the NLRP3 inflammasome plays a role in a variety of human non diabetic kidney diseases and chronic kidney diseases. We [48] and others [49], [50] have shown that NF-κB induces NLRP3 expression and NLRP3 inflammasome activity. In the present study, osthole administration to Prg-IgAN mice resulted in decreased NF-κB activation (Fig. 3A–D), NLRP3 protein expression (Fig. 3E, F), and caspase-1 (p20) activation (Fig. 3E, G) in the kidney. Besides, we also demonstrated that osthole inhibited activation of NLRP3 inflammasome *in vitro* by LPS-activated macrophages (Fig. 6A–F). These data suggest that inhibition of the activation of NF-κB and the NLRP3 inflammasome is involved in the reduction in severe renal lesions in the Prg-IgAN+osthole mice. On the other hand, ROS are also highly implicated in NLRP3 inflammasome activation [48], [51]–[53]. In the present study, Prg-IgAN+osthole mice were found to have greatly reduced production of renal superoxide anion. Consistently, we demonstrated that osthole decreased ATP-induced ROS production and reduced phosphorylation levels of PKC-α and p38 in LPS-primed macrophages (Fig. 7A–C).

Both macrophages [12], [54] and mesangial cells [55], [56] (intrinsic cells in the glomerulus) have been shown to play major pathogenic roles in IgAN. In the present study, characteristic, intense infiltration of macrophages in a pattern of peri-glomerular mononuclear leukocyte infiltration was identified in the kidney of Prg-IgAN mice (Fig. 1D–G and Fig. 4C–F), although infiltration of the cells was not obvious within the glomerulus affected. However, as suggested by Lichtnekert J *et al.* [57], and our preliminary studies, mesangial cells don’t seem to produce NLRP3 to detectable levels. We infer that the inhibitory effect of osthole on the activation of NLRP3 inflammasome more likely arise in the infiltrated macrophages in the kidney of in the Prg-IgAN model. Further investigation on the interactions between ROS, NF-κB, and NLRP3 and precise mechanisms involved in the effects of osthole on the Prg-IgAN model would be helpful in developing osthole as a candidate for therapeutic use before or during the progressive phase of IgAN.

Another potential anti-inflammatory action of osthole seen in this study was its substantially reducing renal MCP-1 levels in Prg-IgAN+osthole mice (Fig. 4A, B). MCP-1 is considered to play a major role in the progression of IgAN, since it recruits mononuclear leukocytes to the lesion sites [58], [59] and since deletion of macrophages ameliorates severe renal inflammatory disorders [60]. Consistent with this, a marked reduction in the severity of the histopathology of renal lesions, including the characteristic infiltration of periglomerular mononuclear leukocytes (macrophages and T cells) (Fig. 4 C–J) was observed in the Prg-IgAN+osthole mice compared to the Prg-IgAN mice. This effect is likely the result of local inhibition of NF-κB activation that causes the production of MCP-1 and resultant mononuclear leukocyte infiltration, in the kidneys of the treated mice. Although the role of macrophages in the glomerulus has been documented in various histological IgAN studies [61]–[63], the Prg-IgAN model showed extremely lower number macrophages infiltrating in the glomeruli affected. We believe that this represents a potential weakness for this mouse model for IgAN.

Activation of the intrinsic pathway of apoptosis, demonstrated by increased renal caspase-9 levels, was observed in the Prg-IgAN mice and this effect was suppressed or markedly inhibited in the Prg-IgAN+osthole mice (Fig. 5E, G), showing that osthole has a protective anti-apoptosis effect in the Prg-IgAN model. Apoptosis is involved in the evolution of IgAN to the progressive form [12], [40], [64], [65] and, in agreement with this, we found that inhibition of apoptosis in the glomerulus of the kidney following osthole administration was associated with relatively mild histopathological renal damage. Since inhibition of apoptosis helps prevent the progression of IgAN [65], inhibition of apoptosis in the kidney may therefore contribute to the beneficial effects of osthole administration on Prg-IgAN.

In the present study, although the *in vitro* experiments suggest that the protective effect of osthole is mediated through an effect on macrophages, this conclusion could not be drawn, because Western blotting was performed in renal cortex lysates, and thus several of the molecules and mechanisms investigated would be operational in the combination of intrinsic glomerular and renal interstitial cells. Besides, to clearly identify the pathogenic role of macrophages in the Prg-IgAN mice, a depletion strategy of the cells *in vivo* [60] is warranted.

Finally, the facts that osthole inhibited renal superoxide anion formation and increased Nrf2 activity in the kidney and prevented renal inflammation in the treated mice suggest it may be a potential drug candidate for preventing IgAN moving from the progression stage (exacerbation) to later clinical and pathological stages.
